# The cyclophilin inhibitor NIM-811 increases muscle cell survival with hypoxia in vitro and improves gait performance following ischemia–reperfusion in vivo

**DOI:** 10.1038/s41598-021-85753-x

**Published:** 2021-03-17

**Authors:** Khairat Bahgat Youssef El Baradie, Mohammad B. Khan, Bharati Mendhe, Jennifer Waller, Frederick O’Brien, Mark W. Hamrick

**Affiliations:** 1 Medical College of Georgia, Augusta University, Augusta, GA USA; 2 Faculty of Science, Tanta University, Tanta, Egypt; 3 Dwight D. Eisenhower Army Medical Center, Fort Gordon, Augusta, GA USA; 4 Department of Cellular Biology & Anatomy, Medical College of Georgia, Augusta University, Augusta, GA 30912 USA

**Keywords:** Biological techniques, Cell biology, Stem cells, Medical research

## Abstract

Acute ischemia–reperfusion injury in skeletal muscle is a significant clinical concern in the trauma setting. The mitochondrial permeability transition inhibitor NIM-811 has previously been shown to reduce ischemic injury in the liver and kidney. The effects of this treatment on skeletal muscle are, however, not well understood. We first used an in vitro model of muscle cell ischemia in which primary human skeletal myoblasts were exposed to hypoxic conditions (1% O_2_ and 5% CO_2_) for 6 h. Cells were treated with NIM-811 (0–20 µM). MTS assay was used to quantify cell survival and LDH assay to quantify cytotoxicity 2 h after treatment. Results indicate that NIM-811 treatment of ischemic myotubes significantly increased cell survival and decreased LDH in a dose-dependent manner. We then examined NIM-811 effects in vivo using orthodontic rubber bands (ORBs) for 90 min of single hindlimb ischemia. Mice received vehicle or NIM-811 (10 mg/kg BW) 10 min before reperfusion and 3 h later. Ischemia and reperfusion were monitored using laser speckle imaging. In vivo data demonstrate that mice treated with NIM-811 showed increased gait speed and improved Tarlov scores compared to vehicle-treated mice. The ischemic limbs of female mice treated with NIM-811 showed significantly lower levels of MCP-1, IL-23, IL-6, and IL-1α compared to limbs of vehicle-treated mice. Similarly, male mice treated with NIM-811 showed significantly lower levels of MCP-1 and IL-1a. These findings are clinically relevant as MCP-1, IL-23, IL-6, and IL-1α are all pro-inflammatory factors that are thought to contribute directly to tissue damage after ischemic injury. Results from the in vitro and in vivo experiments suggest that NIM-811 and possibly other mitochondrial permeability transition inhibitors may be effective for improving skeletal muscle salvage and survival after ischemia–reperfusion injury.

## Introduction

Acute limb ischemia–reperfusion (I/R) injury is a significant clinical concern that can lead not only to local cell death and inflammation in skeletal muscle^1–3^ but also to systemic changes referred to as the systemic inflammatory response^4,5^. The systemic inflammatory response may, in turn, cause multiple organ dysfunction^6,7^. The pathophysiology of I/R injury emanates from two primary processes. First, oxygen deprivation leads to cell death in a subset of the skeletal muscle cell population and second, reperfusion stimulates an inflammatory response that involves the local and systemic release of inflammatory cytokines^8^. Recovery from I/R injury is therefore dependent at least in part on minimizing initial cell death with ischemia and then enhancing survival and recovery of cells that are “potentially salvageable” with reperfusion^8^. Cell survival with ischemia requires modulating levels of oxidative stress, which increases markedly as mitochondria generate abundant H_2_O_2_. Ischemia induces an increase in intracellular Ca++ , which in turn stimulates opening of the mitochondrial permeability transition pore (mPTP) in the mitochondrial membrane^9^. The prolonged opening of the mPTP interrupts the mitochondrial electron transport chain (membrane depolarization), disturbs mitochondrial energy production, and induces production of reactive oxygen species (ROS)^10,11^. These actions release different molecules from the dysfunctional mitochondria that can drive cell death^12,13^. Importantly, increased ROS production is a direct inducer of inflammation by activating NF-ĸß pathway, pro-inflammatory cytokines, and inflammasomes^14^.

Cyclophilin-D is a protein that can modulate structure of the mPTP. Cyclosporine-A (CsA) binds to both the cyclophilin-D component of mPTPs and the cytosolic cyclophilin-A molecules, suppressing the immune response and inhibiting the opening of mPTP^15,16^. N-methyl-4-isoleucine cyclosporine (NIM-811) a non-immunosuppressive cyclophilin inhibitor, is a derivative of Cyclosporine-A (CsA), which binds to Cyclophilin-D thus preventing the development of MPT^17^. NIM-811 has a therapeutic advantage over CsA in that it has no known systemic side effects. This difference between NIM-811 and CsA is due to the fact that NIM-811 cannot bind calcineurin. Calcineurin is a potent regulator of muscle remodeling, skeletal muscle differentiation, regeneration and fiber type specification–all functions that are crucial to muscle development, metabolism and functional adaptation^18^. Previous work has indicated that mPTP inhibition by NIM-811 can enhance cell and tissue recovery following spinal cord injury and traumatic brain injury^19,20^. It has also been found that NIM-811 can preserve renal function and lower circulating inflammatory cytokines following hindlimb I/R injury. Here we tested the hypothesis that NIM-811 can promote cell survival with ischemia directly using primary human cells. We also tested the prediction that the positive effects of NIM-811 and mPTP inhibition on cell survival would translate to muscle-specific changes in inflammation as well as improvements in gait function.

## Results

### Effectiveness of NIM-811 in treating ischemic myoblasts

**Figure 1:**
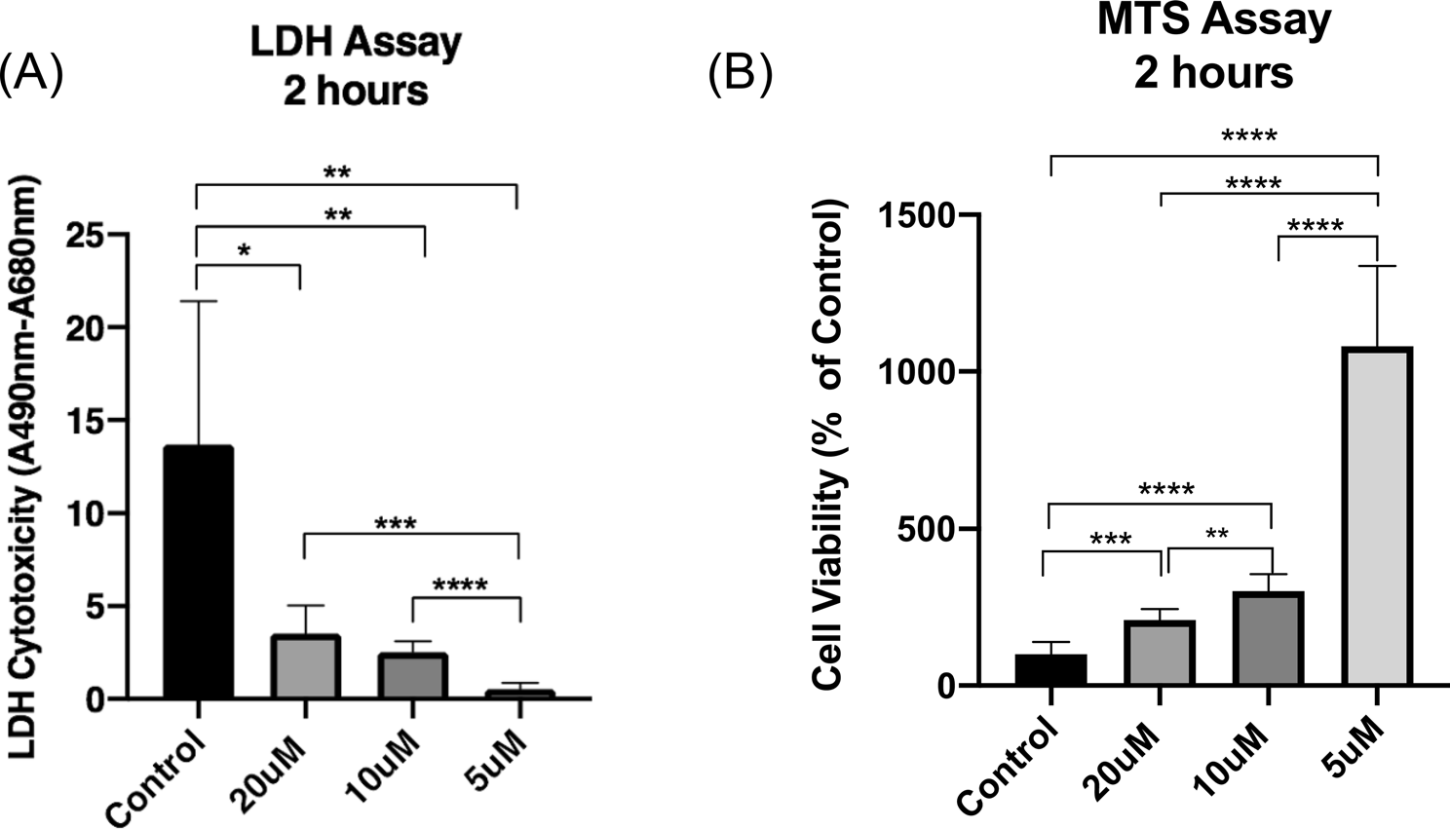
Effects of NIM-811 treatment on ischemic human myoblasts. Ischemic Human myoblasts were treated with (0–20 μM) NIM-811 for 2 h following the exposure to hypoxia for 6 h. Data shown are for cell viability measured by MTS assay (**A**) and cell toxicity in different groups as determined by LDH assay (**B**). Data are expressed as mean ± SD (n = 6). ****P = 0.0001, ***P = 0.0005, **P = 0.0022 and *P = 0.01.

We evaluated the effects of NIM-811 on the viability of ischemic human myoblasts by treating cells with 0–20 μM of NIM-811 for 2 h. MTS assays showed that there was a statistically significant, dose-dependent, difference in cell number between the control group and treated groups (Fig. 1A). Furthermore, the LDH data indicate that 5 μM NIM-811 was significantly more effective in ameliorating ischemic injury compared to higher doses (10–20 μM) of NIM-811 (Fig. 1B).

### Identification of elastic lasso tourniquet-induced ischemia–reperfusion model

**Figure 2:**
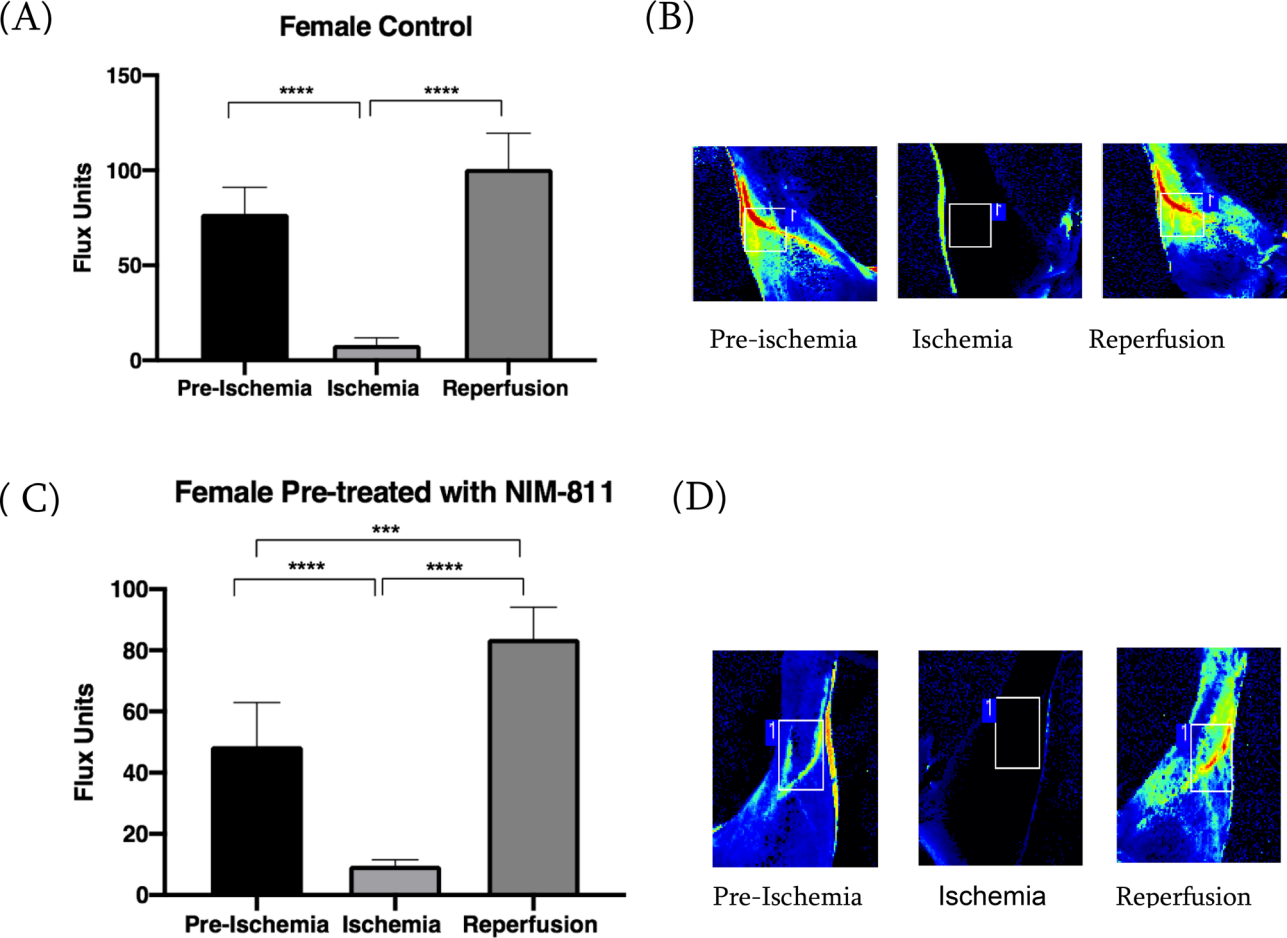
Laser Doppler imaging of female mice models of hindlimb ischemia. (**A**) Blood flow measurements of ischemic left hindlimbs in female vehicle (control)-treated mice subjected to 90 min ischemia the hindlimb baseline (pre-ischemia) flux, as well as the reperfusion flux, were significantly ****P = 0.0001 higher than ischemic flux. (**B**) Representative Laser Doppler images in control mice during baseline, ischemia, and reperfusion. (**C**) Blood flow measurements of ischemic left hindlimbs in female mice treated with NIM-811 subjected to 90 min ischemia the hindlimb baseline (pre-ischemia) flux as well as the reperfusion flux were significantly ****P = 0.0001 higher than ischemic flux. Moreover, the reperfusion flux was significant ***P = 0.0006 vs. baseline (pre-ischemia). (**D**) Representative Laser Doppler images in female mice treated with NIM-811 during baseline, ischemia, and reperfusion.

**Figure 3:**
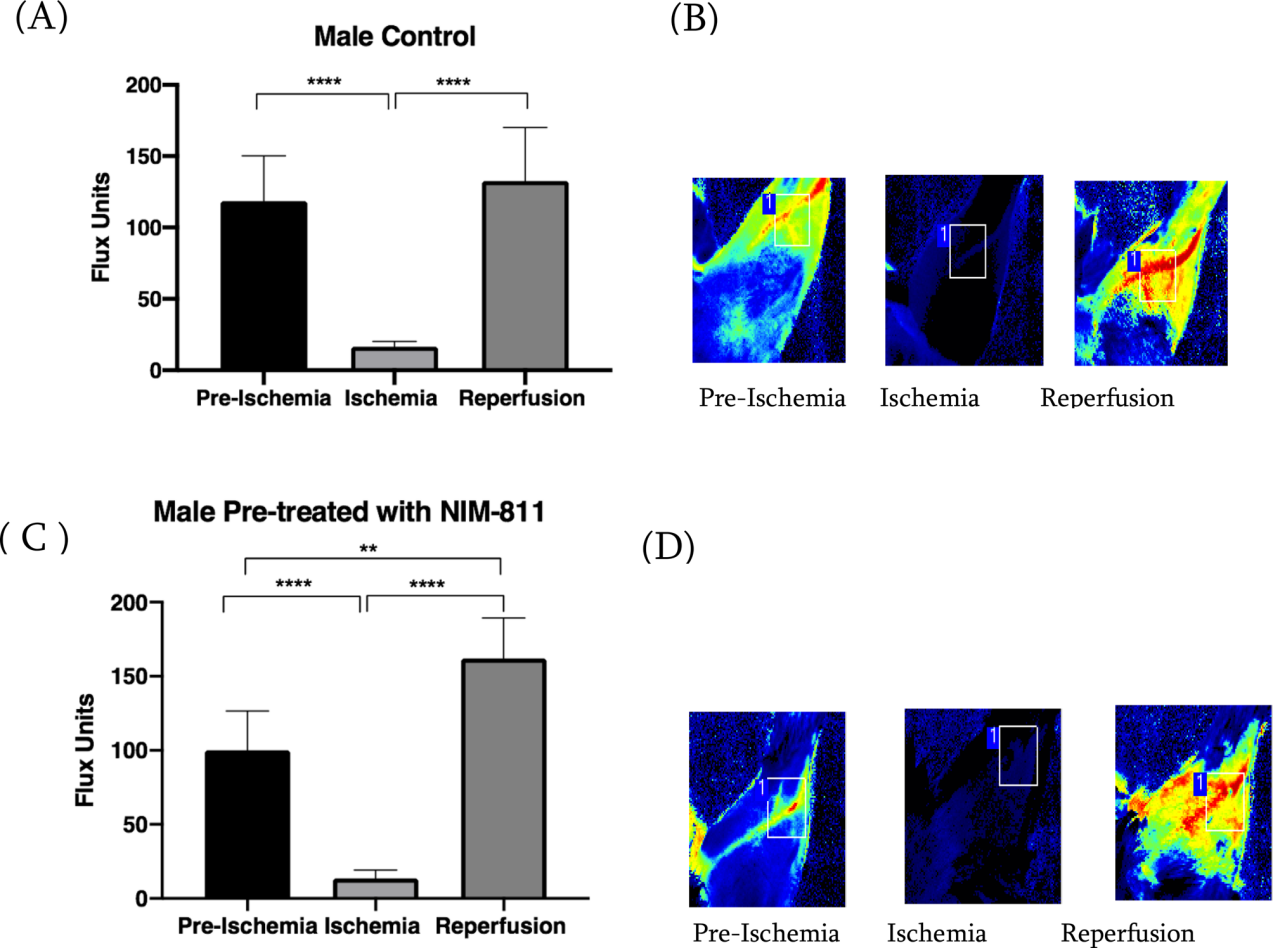
Laser Doppler imaging of male mice models of hindlimb ischemia. (**A**) Blood flow measurements of ischemic left hindlimbs in male vehicle (control)-treated mice subjected to 90 min ischemia the hindlimb baseline (pre-ischemia) flux, as well as the reperfusion flux, were significantly ****P = 0.0001 higher than ischemic flux. (**B**) Representative Laser Doppler images in control mice during baseline, ischemia, and reperfusion. (**C**) Blood flow measurements of ischemic left hindlimbs in male mice treated with NIM-811 subjected to 90 min ischemia the hindlimb baseline (pre-ischemia) flux, as well as the reperfusion flux, was significantly ****P = 0.0001 higher than ischemic flux. Moreover, the reperfusion flux was significant **P = 0.0006 vs. baseline (pre-ischemia). (**D**) Representative Laser Doppler images in male mice treated with NIM-811 during baseline, ischemia, and reperfusion.

Inducing limb ischemia in a rodent model using a tourniquet is a well-established technique^21–23^. A laser doppler imager was used to measure blood flow in the ischemic left limb to verify the muscle ischemia. Once we applied the tourniquet, the blood flow remained steady during the 90 min of ischemia to about 1% of the baseline. At the beginning of the reperfusion phase, once the tourniquet is released, an increase in the blood flow to approximately 10% of the baseline was observed in the control group. However, in the NIM-811 treated group, the tourniquet release led to an increase in blood flow to approximately 50% of the baseline in both female (Fig. 2A–D) and male (Fig. 3A–D) mice. Pretreatment with NIM-811 (10 mg/kg) before ischemia significantly improved blood flow during reperfusion.

### Mice treated with NIM-811 had enhanced limb function and performance

There were no statistically significant differences between male and female animals for distance, speed, or gait scores (Table 1). However, both the speed (P = 0.0051) and the limb function (gait score; P = 0.0096) were significantly enhanced with NIM-811 treatment (Table 2).

**Table 1:** Descriptive statistics of gait measures by sex and treatment group and the two-factor ANOVA F-test for the sex by treatment interaction.

Variable	Sex	Treatment	Mean	SD	F	p-value
Distance	Female	Control	11.49	4.12	0.36	0.5580
		NIM-811	16.31	4.45		
	Male	Control	11.05	3.98		
		NIM-811	13.42	5.56		
Speed	Female	Control	0.12	0.09	0.21	0.6566
		NIM-811	0.27	0.08		
	Male	Control	0.06	0.03		
		NIM-811	0.26	0.21		
Gait score	Female	Control	1.80	0.45	0.00	1.0000
		NIM-811	2.80	0.84		
	Male	Control	1.60	0.89		
		NIM-811	2.60	0.89

**Table 2:** Two-factor main effects ANOVA model results on gait measures.

Gait measure	Variable	Level	Mean	SD	F	p-value
Distance	Sex	Female	13.90	4.78	0.69	0.4178
		Male	12.23	4.73		
	Treatment	Control	11.27	3.83	3.22	0.0905
		NIM-811	14.87	4.99		
Speed	Sex	Female	0.19	0.11	0.38	0.5484
		Male	0.16	0.18		
	Treatment	Control	0.09	0.07	10.31	0.0051
		NIM-811	0.26	0.15		
Gait score	Sex	Female	2.30	0.82	0.34	0.5675
		Male	2.10	0.99		
	Treatment	Control	1.70	0.67	8.50	0.0096
		NIM-811	2.70	0.82		

### Histological changes in skeletal muscle

**Figure 4:**
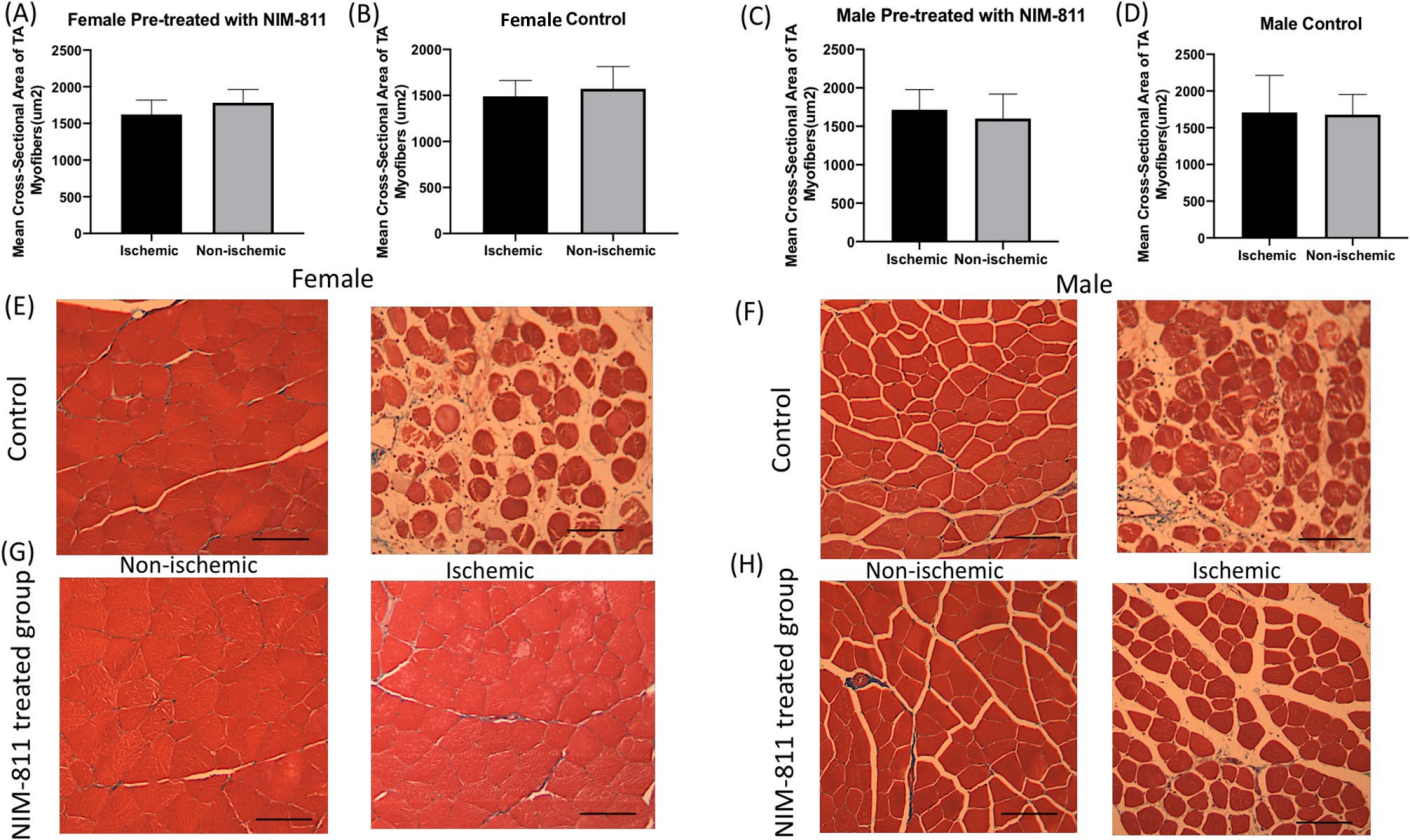
Histology of muscle tissue. (**A**–**D**) Morphometric analysis of muscle fiber cross-sectional area of the TA muscles intact fibers in female and male mice does not differ significantly between the ischemic limbs of vehicle (control) and NIM-811 treated mice. (**E**–**H**) Hematoxylin and eosin (H&E)/ trichome sections of TA muscle fibers from the ischemic limbs of vehicle-treated female and male mice show some shrinkage, necrosis, and irregular morphology compared to non-ischemic limb from the same group. The muscle fiber morphology was more regular, and staining was more uniform from the ischemic limbs of NIM-811 treated mice, most of the muscle cell was still located in the muscle membrane, and degeneration less frequent compared to the non-ischemic limb from the same group. Scale bar = 100 μm.

TA muscle cross-sectional area of the intact fibers did not show significant mean differences between ischemic and non-ischemic limbs within control and treated group of both sexes (Fig. 4A–D). Histological analysis of muscle cell morphology in both male and female animals does, however, show that muscles from the ischemic limbs of control (vehicle) treated mice display a disordered, irregular muscle fiber morphology (Fig. 4E,F). In contrast, muscle fibers from the ischemic limbs of NIM-811 treated mice are more regular, and degeneration is not so obvious (Fig. 4G,H).

### 4-HNE staining for lipid peroxidation as a marker of oxidative stress

**Figure 5:**
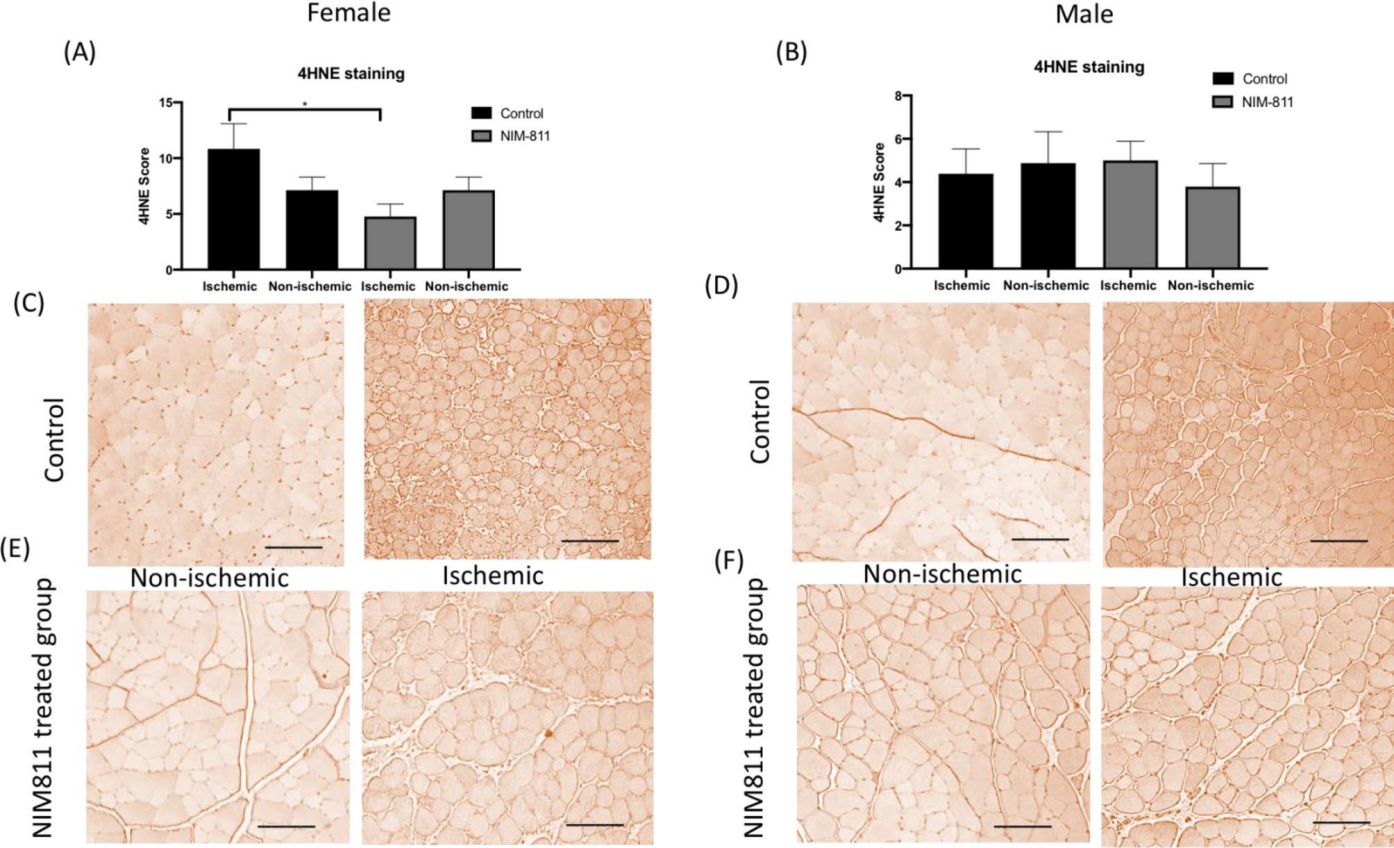
Staining for lipid peroxidation in muscle. (**A**,**B**) Qualitative difference in lipid peroxidation (4-HNE) staining in TA skeletal muscle from the ischemic and non-ischemic hindlimbs of mice. Lipid peroxidation decreased significantly in muscle from the ischemic limb of female mice treated with NIM-811. *P < 0.05. (**C**–**F**) Representative images of TA muscle stanning with lipid peroxidation marker 4-HNE showed dense staining in the ischemic limbs of female vehicle (control) compared to ischemic muscle of NIM-811 treated group. Scale bar = 50 μm.

NIM-811 significantly reduced (P < 0.05) 4-HNE staining in muscles from ischemic limbs of female mice treated with NIM-811 (Fig. 5A); however, in male mice there, was no significant difference between the male ischemic control muscle fibers compared to the ischemic NIM-811 treated muscle fibers (Fig. 5B). Immunohistochemistry analysis of muscle cells in both male and female animals does show that muscles from the ischemic limbs of control (vehicle) treated mice display a higher immunoreactivity to 4-HNE compare to ischemic limb from the NIM-811 treated group in female mice (Fig. 5C,E). However, that was not the case in muscle fibers from the ischemic limbs of control (vehicle) treated mice compared to the ischemic limb from the NIM-811 treated group in male mice (Fig. 5D,F).

### Cytokine changes in serum and skeletal muscle

**Figure 6:**
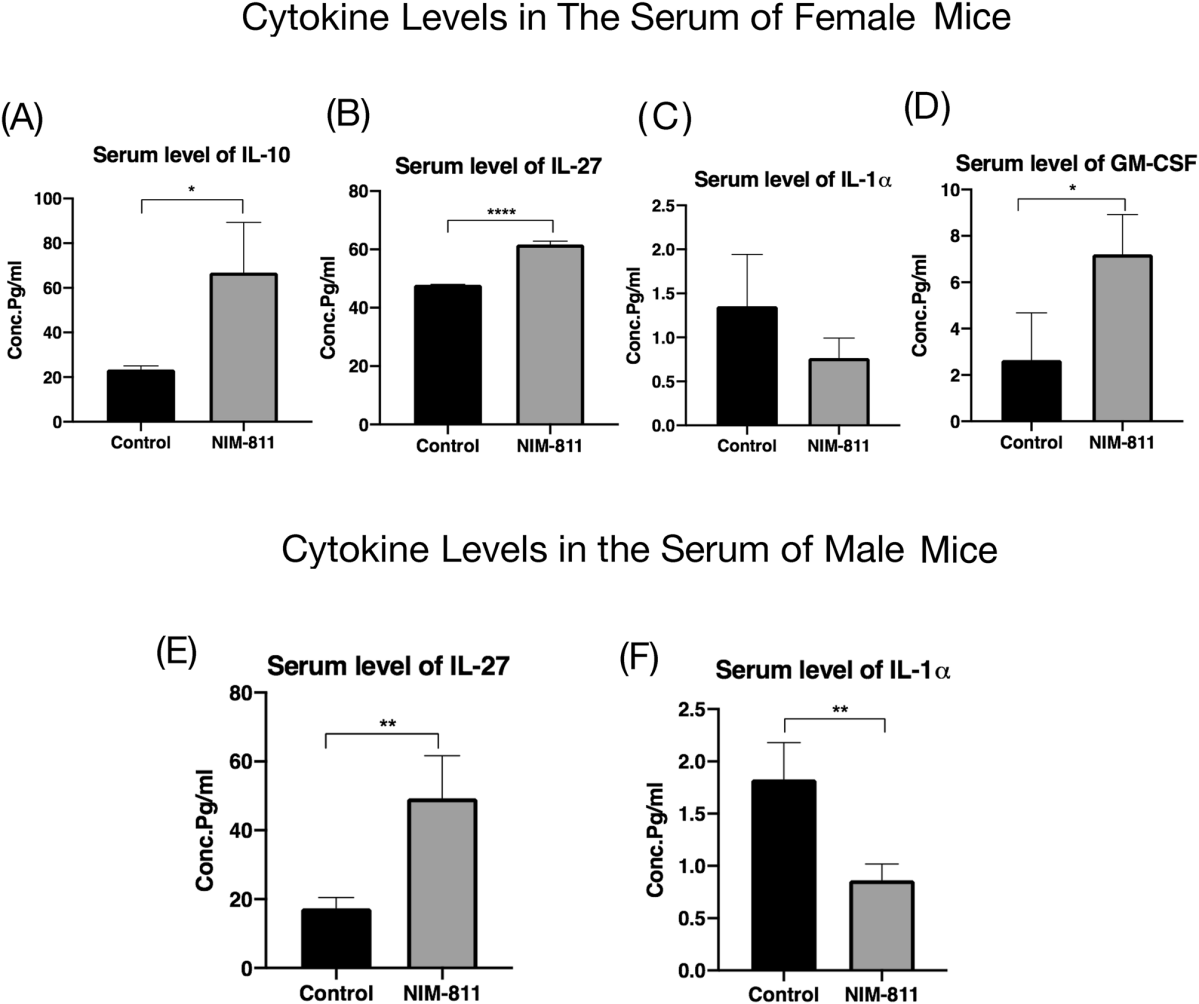
Serum cytokine levels in female and male mice after hindlimb ischemia–reperfusion and treatment with NIM-811. (**A**–**C**) Serum IL-10, IL-27 and GM-CSF levels increased significantly in the serum of female mice treated with NIM-811 compared to control croup. (**D**) Serum IL-α level decreased significantly in the serum of female mice treated with NIM-811 compared to control group. (**E**) Serum IL-27 level increased significantly in the serum of male mice treated with NIM-811 compared to control croup. (**F**) Serum IL-α level reduced in the serum of male mice treated with NIM-811 compared to control croup. *P < 0.05, **P < 0.01 and ***P < 0.001.

There was a significant elevation in serum levels of IL-10, IL-27, and GM-CSF, and a significant decrease in IL-1α, in the female mice pre-treated with NIM-811 compared to the control group at 24 h of reperfusion (Fig. 6A–D). There was a significant elevation in serum levels of IL-27 and a considerable reduction in IL-1α in the male mice pre-treated with NIM-811 compared to their control group at the same time point (Fig. 6E,F).

**Figure 7:**
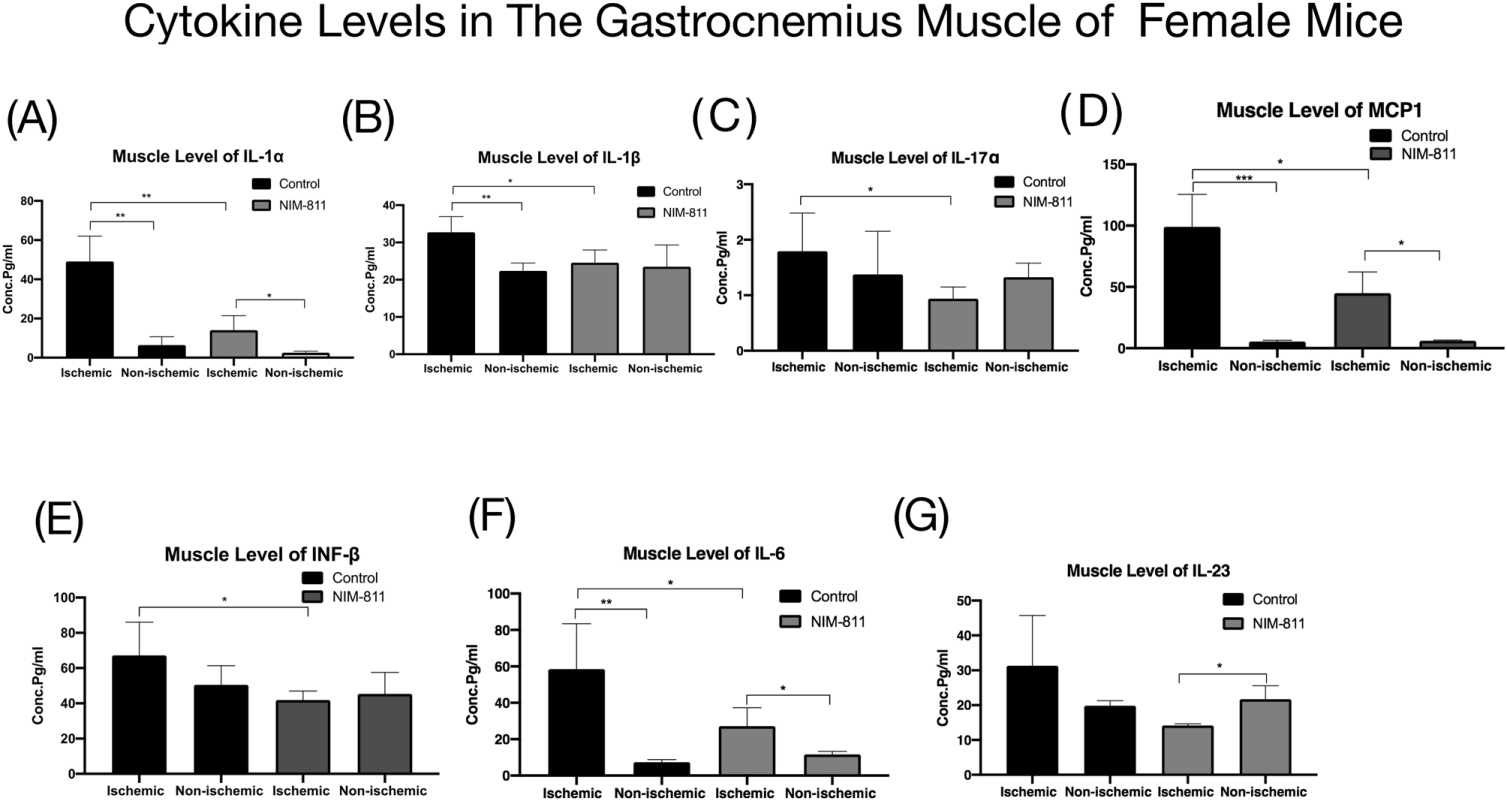
Tissue cytokine levels in female mice after hindlimb ischemia–reperfusion and treatment with NIM-811. (**A**–**F**) Tissue IL-1α, IL-1β, IL-17α, MCP 1, INF-β, and IL-6 levels decreased significantly in the ischemic muscle of female mice treated with NIM-811 compared to the ischemic muscle of the control group. (**G**) Tissue IL-23 level decreased significantly in the ischemic muscle of female mice treated with NIM-811 compared to the non-ischemic muscle of the same group. ***P < 0.001, **P < 0.01 and *P < 0.05.

**Figure 8:**
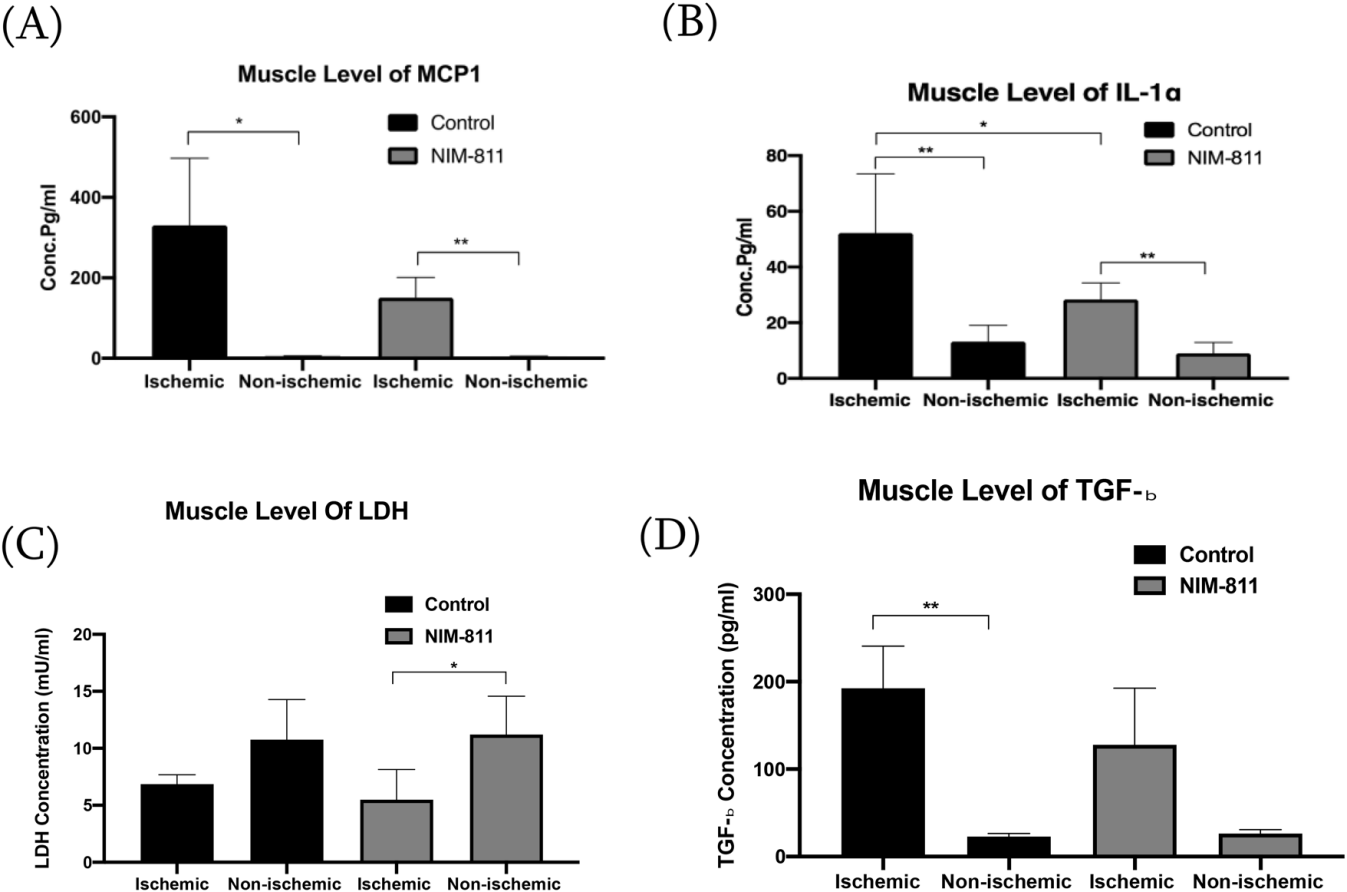
Tissue cytokine levels in male mice after hindlimb ischemia–reperfusion and treatment with NIM-811 and LDH and TGF-β protein levels in gastrocnemius muscle of female mice. (**A**,**B**) Tissue IL-1α, and MCP1 levels decreased in the ischemic muscle of male mice treated with NIM-811 compared to the ischemic muscle of the control group. (**C**) LDH concentration in the protein lysate from gastrocnemius muscle in female mice Indicates significant differences between ischemic muscle of the NIM-811 treated group compared to non-ischemic muscle of the same group. (**D**) Acid activated protein lysates from gastrocnemius muscle samples were assessed for TGF-β1 by ELISA and compared to a standard curve in female mice indicates significant differences between ischemic muscle of control group compared to non-ischemic muscle of the same group. **P < 0.01 *P < 0.05.

Cytokine data from muscle lysates of the ischemic limbs of female mice treated with NIM-811 showed significantly lower levels of pro-inflammatory factors IL-1α, IL-1B, IL-17α, MCP-1, INF-B, and IL-6 (Fig. 7A–F) compared to muscle lysates from the ischemic limbs of vehicle-treated mice. Additionally, muscle lysates from the ischemic limbs of female mice treated with NIM-811 showed significantly lower levels of IL-23 compared to the non-ischemic limb of the same group (Fig. 7G). Similarly, male mice treated with NIM8-11 showed lower levels of MCP-1 and IL-1α in muscle lysates from the ischemic limb compared to lysates from ischemic limbs of vehicle-treated mice (Fig. 8A,B).

### LDH levels in muscle tissue

The tissue LDH concentration was lower at the ischemic gastrocnemius muscle compared with non-ischemic muscle in the female control group. However, the pretreatment with NIM-811 leads to a significant decrease in the LDH concentration in ischemic muscle compared to the non-ischemic muscle from the same group (Fig. 8C).

### TGF-β1 protein expression in gastrocnemius muscle

ELISA analysis of ischemic muscle lysates showed a significant increase in the TGF-β1 level compared to the non-ischemic muscle in the female control group. This increase between the ischemic and non-ischemic muscle was not significant in the female mice treated with NIM-811 (Fig. 8D).

## Discussion

Acute limb ischemia can cause severe pain, poor limb function, and even compromise patient survival^24^. Skeletal muscle is highly susceptible to ischemic insult, and ischemia may cause irreversible muscle damage depending on the duration of impaired perfusion^25^. Limb reperfusion may, in turn, cause further muscle damage with the release of myoglobin, LDH, and other intracellular muscle contents. Systemic immune response and mitochondrial dysfunction are the major contributors to loss of myocytes during myocardial ischemia and subsequent reperfusion^25^. A variety of pharmacological therapies have been proposed for the treatment of limb IR injury. CsA was widely investigated in previous studies during ischemia-reperfusion^17^. The main limitation of this drug is that it is non-specific. Besides binding to cyclophilin D, a key component of the mitochondrial transition pore, it also binds to cytosolic cyclophilin A. Cyclophilin A has several molecular targets within the cellular survival/death pathways that lead to a wide range of adverse effects such as immunosuppression, nephrotoxicity, and hepatotoxicity^26,27^. For these reasons, finding a safe and effective small molecule that can prevent ischemia–reperfusion injury is essential. In the present study, we used a more specific and potent inhibitor of the MPT pore named *N*-methyl-4-isoleucine-cyclosporin (NIM-811). NIM-811 is non-immunosuppressive and does not interact with the cytosolic cyclophylin A^28^. We report here that inhibition of the MPT pore can protect ischemic human myoblasts in an in vitro model of ischemia–reperfusion. MTS assay showed that there was a statistically significant difference in cell numbers between all treated groups and the control group. Furthermore, the LDH assay results suggest that NIM-811 was highly effective in ameliorating the ischemic injury compared to those treated with vehicle.

Muscle fiber necrosis, disintegration and edema were detectable in the muscles from ischemic limbs of vehicle (control) treated mice whereas muscles from the ischemic limbs of NIM-811 treated mice showed less obvious pathological changes. This may explain the greater speed and the limb function (gait score) with NIM-811 treatment compared to the vehicle-treated group. Staining for lipid peroxidation demonstrated that NIM-811 treatment significantly reduced levels of oxidative stress in skeletal muscle from ischemic hindlimbs. Since NIM-811and CsA cannot act as direct antioxidants because they lack the required chemical structure for that action, they may indirectly, via the reduction in oxidative damage, maintain mitochondrial homeostasis. Fewer free radicals and less oxidative damage were observed as a result. Moreover, we observed greater reperfusion in vivo with NIM-811 treatment, suggesting that this drug may preserve the microvasculature response, which might positively impact long-term graft function after limb reperfusion^29^. Importantly we also observed significant sex differences in the response of muscle tissue to NIM-811 following ischemia–reperfusion. Specifically, NIM-811 treatment significantly reduced 4-HNE staining in muscles from females but not in males, which was also reflected in sex differences in cytokine levels (see below). There are now documented examples of sex differences in MPT with ischemia-reperfusion^30^^.^ For example, cardiac mitochondria of females are thought to be less susceptible to MPT than male mitochondria, whereas female brain mitochondria may be more susceptible to MPT^30^. Our data suggest that small molecules such as NIM-811 that target MPT may be more effective in females in the setting of ischemia–reperfusion injury.

One of the systemic complications following limb IR is the local inflammatory response that can contribute to tissue damage^31^. Different inflammatory mediators and proinflammatory cytokines are thought to be released during this process. In our experiments, IL1-α showed a considerably lower value in the serum of the NIM-811 treated-group in male mice, and IL1-α and IL1-β were both reduced in the female mice with NIM-811 treatment. Previous work has shown that the pro-inflammatory cytokine IL-1 is involved in the pathogenesis of a wide range of inflammatory diseases^32^. IL-1 blockade is the standard of care for the treatment of “autoinflammatory diseases”, a family of conditions characterized by dysfunction of monocytes/macrophages and recurrent bouts of debilitating inflammation^33^. Serum levels of several anti-inflammatory cytokines such as IL-10 and GM-CSF were significantly elevated in NIM-811 treated female mice, and IL-27 was significantly elevated with NIM-811 treatment in both sexes. These findings indicate that enhanced muscle structure and function in the ischemic limb with NIM-811 treatment had not only local but systemic effects, suggesting that NIM-811 may promote cellular function in multiple organ systems following ischemic injury.

We also observed marked changes in the levels of inflammatory cytokines in skeletal muscle tissue lysates with NIM-811 treatment. Pro-inflammatory cytokines IL-17α, IL-23, IL-6 as well as INF-β were significantly lower in gastrocnemius muscles from ischemic limbs of NIM-811 treated female mice. Moreover, the lower IL1-α serum levels of NIM-811 treated male mice, and lower serum IL1-α and IL1-β in female NIM-811 treated mice, corresponded to similar decreases in skeletal muscle IL1-α and IL1-β levels with NIM-811 treatment. MCP-1 level was also significantly lower in ischemic muscles from NIM-811 treated mice compared to ischemic muscles from vehicle-treated mice. In skeletal muscle, MCP-1 is known to promote macrophage infiltration after (severe) tissue damage and represents a molecular link in the crosstalk between adipose tissue and skeletal muscle^34–36^. Previous findings have documented elevated MCP-1 in response to different stressors, and our data showing lower MCP-1 in muscle with NIM-811 is consistent with an overall decrease in the inflammatory response^37–39^. TGF-β1 is a major contributor to fibrosis after injury in various tissues and is increased after injury in regenerating muscle^40^. We found no significant effect on muscle TGF-β1 with NIM-811 treatment. Differentiating between total and active forms of TGF-β1 present in the muscles of each experimental group may be needed to provide additional, more relevant information for comparison.

Our study demonstrated that NIM-811 was capable of enhancing the levels of anti-inflammatory cytokines in muscle tissue, decreasing levels of pro-inflammatory cytokines, and improving limb function after ischemia–reperfusion. These findings are consistent with previous work demonstrating positive effects of NIM-811 on cell survival and tissue function following spinal cord injury and traumatic brain injury^19,20^. Together these results indicate that NIM-811 or similar molecules that target the mPTP could be useful in the trauma setting, and perhaps improve patient outcomes following the injury to multiple organs and tissues.

## Material and methods

### Hypoxic cell culture and treatment of primary human myoblasts

Primary human skeletal myoblasts purchased from ThermoFisher Scientific (Catalog # A11440) were cultured as monolayers in Dulbecco's Modified Eagle Medium (DMEM/High Glucose), supplemented with 5% (v/v) fetal bovine serum (FBS) and 1% (v/v) penicillin/streptomycin (Pen-Strep) as we described previously^41^. The medium was changed after 24 h to discard non-adherent cells. Media was changed every 2 days, and the cells were expanded until passage 4 to 6. The expanded myoblast cells were then seeded in 48-well plates at a density of 7000 cells/well and allowed to attach and grow for 24 h. The culture medium was changed the following day to DMEM containing 4.5 g/L glucose without l-glutamine and phenol red. Myoblasts were then exposed to hypoxia condition (1% O_2_ and 5% CO_2_) for 6 h.

A stock solution of N-methyl-4-isoleucine cyclosporine NIM-811 (NIM-811; MedChemExpress, NJ, USA) was prepared by dissolving in DMSO at a concentration of 1 mM. As we described previously^41^, different volumes of the stock solution were added to myoblast cultures during the last 20 min of the hypoxia-period to obtain (0–20 µM) final concentrations of NIM-811 in the culture medium (phenol red-free DMEM supplemented with 1% FBS). At the end of the hypoxia period the myoblast cultures were transferred to a normoxic incubator for an additional 2 h.

### Assaying viability and proliferation of ischemic primary human myoblasts

After the ischemic human myoblasts were treated with NIM-811 as detailed above, the MTS and LDH assays were performed as we showed previously to assess the tissue damage after I/R^41^. In brief, in each well the ischemic myoblasts after the removal of the culture medium, were mixed with 100 μL of 3-(4,5-dimethylthiazol-2-yl)-5-(3-carboxymethoxyphenyl)-2-(4-sulfophenyl)-2H-tetrazolium, inner salt (MTS, Promega Corporation, FL, USA). The optical density (OD) was measured at 490 nm using a microplate reader. Each group was run in sextuplicate. For LDH assay the culture media was collected from each treatment group and the absorbance measured at 490 nm and 680 nm. The LDH activity was determined after the subtraction of the 680 nm absorbance value (background) from the 490 nm absorbance. % Cytotoxicity = (Compound-treated LDH activity − spontaneous LDH Activity)/(Maximum LDH activity − spontaneous LDH activity) × 100 was calculated as the percentage of cytotoxicity. The data for each group were obtained from sextuplicate experiments and shown as mean percentages.

### In vivo ischemia model

Animal care and experimental procedures complied with “Principles of Laboratory Animal Care” (*Guide for the Care and Use of Laboratory Animals*, National Institutes of Health Publication No. 86-23, Revised 1996) and were approved by the Augusta University IACUC. All animal experiments and analysis were performed in accordance with the ARRIVE guidelines. Twenty CD-1 mice (28–35 g) (Envigo RMS, Inc. Indianapolis, IN; 10 males and 10 females), were allowed access to water and chow ad libitum with a 12-h:12-h light–dark cycle and the room temperature was kept constant between 24 and 26 °C. The 10 male and 10 female mice were randomly allocated into two groups of five mice each; Group 1 (Control group) was subjected to all operative procedures (90 min single hindlimb ischemia and 24 h perfusion). The animals received boluses of Phosphate-buffered saline (PBS) with the same concentration of DMSO used in the treated group, 10 min before reperfusion, and 3 h into the reperfusion period. Group 2 (NIM-811 treated group) were subjected to the same procedure of ischemia–reperfusion. The NIM-811 treated group received boluses of NIM8-11 at a concentration of (10 mg/kg) 10 min before reperfusion, and 3 h into the reperfusion period. A solution of NIM-811 dissolved in DMSO and diluted in PBS solution was administered as intraperitoneal (i.p.) injection in a final concentration of 10 mg/kg.

### Limb perfusion measurement

Isoflurane was delivered by face mask, 2% for induction, and 1% for maintenance, along with continuous oxygen at 2 l/min while the mice were kept on a heating pad to maintain the body temperature at 37 °C. Following induction of anesthesia, the fur was removed from the left hindlimb with a hair removal cream and the skin cleaned with 70% ethanol. The Laser Doppler imager (Moor Instruments, Wilmington, DE) was used to assess limb perfusion^42,43^. The animal remained on the warming table under isoflurane anesthesia during the 90 min of ischemia. The Laser Doppler source was mounted on a movable rack 10 cm above the mouse hindlimb, and blood movement was detected and processed by the laser beam (780 nm) to provide a computerized, color-coded image. Mean flux values representing tissue perfusion were calculated from the relative flux (in U/cm^2^) in the areas corresponding to the plantar aspect of the hindlimb using image analysis software (Laser Doppler perfusion measure, V3.08, Moor Instruments). Baseline images were obtained from each mouse immediately after the induction of anesthesia and hair removal to assess the limb ischemia. Ischemia was induced on the proximal thigh with ORBs 4.5 oz (American Orthodontics, Sheboygan, WI). This diameter was chosen based on measurements of the thigh of CD-1 mice (28–35 g) mice, and the previous study uses this tourniquet to induce limb ischemia in a rodent model^23^. Another Laser Doppler image was obtained 30 min into the procedure to assess limb ischemia. Data were expressed as percent basal perfusion in the limbs.

Mouse hindlimb was scanned under isoflurane anesthesia at three intervals: (1) right after the induction of anesthesia (baseline), (2) 30 min after applying of rubber band ischemia and (3) right after the removal of the rubber band and start of reperfusion. The NIM-811 treated group received boluses of NIM-811 at a concentration of (10 mg/kg) 10 min before reperfusion, and 3 h into the reperfusion period. Control mice received boluses of PBS with the same concentration of DMSO, 10 min before reperfusion, and 3 h into the reperfusion period.

### Video recording and gait score analysis

After the 90 min of the ischemic period, mice were allowed to recover from anesthesia. Mice were then videotaped walking on trackway and their gait score was recorded. Mice were filmed using Digital Video Camcorder (GoPro Hero8). The video recordings were analyzed frame-by-frame at 15 f/s using the Kinovea version 0.8.24. The procedure included four steps: (1) Kinovea frame calibration; (2) Images digitization; (3) Export of distance traveled as well as speed data to a spreadsheet; (4) Data analysis. Semi-quantitative assessments of limb function after applying the ischemia/reperfusion were performed once the mice were recovered from anesthesia. Limb function was assessed using the clinical use score (Tarlov scale) as 0 = no movement; 1 = barely perceptible movement, no weight-bearing; 2 = frequent and vigorous movement, no weight-bearing; 3 = supports weight, may take 1 or 2 steps; 4 = walks with the only mild deficit; 5 = normal but slow walking and 6 = full and fast walking^44,45^.

### Tissue and blood processing

After 24 h of reperfusion, the animals were euthanized and about one mL of whole blood was collected from each mouse heart. The blood kept was on ice for 2–3 h, centrifuged for 30 min at 1000 rpm, and serum collected for cytokine array. Skeletal muscle tissue samples collected at the time of death from both hindlimbs included tibialis anterior (TA) and gastrocnemius muscles. TA muscles were fixed in 10% neutral buffered formalin and prepared for histology. Gastrocnemius muscles were flash-frozen in liquid nitrogen and stored at − 70 °C for lactate dehydrogenase assay (LDH), cytokine array, and enzyme-linked immunosorbent assay (ELISA).

TA muscles were paraffin-embedded and cross-sections cut at the 2–3-μm thickness and stained with Masson trichrome. Stained slides were examined under optical microscopy at 20× magnification. Muscle fiber cross-sectional area (CSA) was measured using Image J software^46^. Fifty random fields were analyzed using a cool color camera and RS image software program (Ocular Image Acquisition Software, QImaging, Canada). Muscle fibers were scored as uninjured as having well-defined borders, consistency of texture, and uniformity throughout the fiber without holes or breaks. Total areas are measured using the “analyze” and “measure” functions which will report area, minimum, maximum, and the mean.

### Immunohistochemistry study

Paraffin-embedded serial sections of TA muscle were deparaffinized, rehydrated, blocked in with 5% nonfat dried milk, and incubated with immunoglobulin G-purified polyclonal rabbit antibodies specific for 4-HNE overnight at 4 °C. Immunoreactivity was detected using a secondary biotinylated goat anti-rabbit antibody (1:200). Avidin/biotinylated horseradish peroxidase kit (Pierce, Rockford, IL) was used to visualize the interactions between the primary and secondary antibodies. Immunostaining procedure in the absence of primary antibody was used to eliminate the non-specificity immunoreactivity that might be a result of nonspecific interactions of the secondary antibody.

### LDH assay for assessment of the cellular injury

Degree of cellular injury was assessed based on the activity of LDH in protein extracts from gastrocnemius muscle in ischemic and non-ischemic limb in both control and NIM-811 treated groups as showed previously^47^. LDH level was assessed spectrophotometrically by monitoring NADH oxidation at an absorbance at 450 nm (Cytotoxicity Detection Kit MAK066, Sigma-Aldrich), according to the manufacturer’s instructions.

### Cytokine antibody array

Cytokine Bead Array Mouse Inflammation 13-Plex Panel (Bio-Legend, San Diego, CA, USA) was used for estimating the differential amount of cytokines in pooled protein extracts from gastrocnemius muscle as well as serum, accordingly to the manufacturer’s instructions.

### Determination of TGF-beta1 protein

Total TGF-beta1 was determined by ELISA, the kit according to manufacturer’s instructions (R&D Systems, Minneapolis, MN, USA), on the gastrocnemius muscle from both limbs of NIM-811 treated group as well as the control group in male and female mice. Briefly, a 500 μl of a solution containing 1% Triton X-100, 20 mM Tris/Hydrochloric acid (HCl) pH 8.0, 137 mM sodium chloride (NaCl), 10% glycerol, 5 mM ethylenediaminetetraacetic acid (EDTA), 1 mM phenyl-methyl-sulfonyl-urea, 1% aprotinin, and 15 μg/ml leupeptin was used to homogenized a 10–20 mg of gastrocnemius muscle. To activate latent TGF-beta 1 activity 1 N HCl 1:1 was added to 1 μg total protein. Optical density was determined within 30 min of adding stop solution by using a microplate reader set at 450 nm^48^.

### Statistical analysis

Statistical analysis was performed using SAS 9.4 and statistical significance was assessed using an overall alpha level of 0.05. Means and standard deviations within sex, limb, treatment, and measurement time (where appropriate) were determined for all outcome measures. All post hoc pairwise comparisons were performed using a Bonferroni adjustment to the overall alpha level for the number of pairwise comparisons made. Repeated measures mixed models were used to examine differences between treatments (NIM-811 versus control) by sexes and limbs (ischemic versus control). For LDI blood flow, a repeated measures mixed model was used to examine differences over time (pre-ischemia, during ischemia, reperfusion) between treatments and sexes on the ischemic limb. To examine whether differences in distance, speed, and gait score between treatment groups and sex, two-factor ANOVA was used. Main effects of treatment group and sex as well as the two-factor interaction were included in each ANOVA model.
